# Acute Surgery vs. Non-union Surgery of Displaced Midshaft Clavicle Fractures: A Case-control Study

**DOI:** 10.7759/cureus.5480

**Published:** 2019-08-25

**Authors:** Michael Marsalli, José T Rojas, Maximiliano Barahona

**Affiliations:** 1 Orthopaedic Department, Clínica Universidad De Los Andes, Santiago, CHL; 2 Orthopaedic Department, Hospital San José, Santiago, CHL; 3 Orthopaedic Department, Hospital Clinico Universidad De Chile, Santiago, CHL

**Keywords:** clavicle fracture, clavicle non-union, clavicle surgical treatment, clavicle non-union surgery, midshaft clavicular fractures, clavicle treatment

## Abstract

Introduction

There is a lack of information about the results of surgical treatment and complications in midshaft clavicle fracture non-unions. Our hypothesis was that there is no difference in functional outcomes between the surgical treatment of an acute displaced middle-third clavicle fracture and the surgical treatment of a chronic symptomatic non-union of a displaced middle-third clavicle fracture.

Methods

This was a case-control study. Fourteen cases were considered with a displaced midshaft clavicle fracture, initially treated non-surgically, but which developed symptomatic non-union and required surgical treatment. The control group was a cohort of 18 patients with a displaced midshaft clavicle fracture, who had surgical treatment in an acute setting (<3 weeks). Our cases had a median follow-up of 77 months and were retrospectively analyzed. All those in the control group had a 12-month prospective follow-up evaluation. The variables measured were Constant score, time to discharge to work, and bone union rate.

Results

The median Constant score at final follow-up for surgically treated non-unions was 87.5 (control group 84.5, p > 0.05). The median time to complete return to work was 3.2 months in the control group and 9.7 months in the case group (p=0.001). Hundred percent of those patients who were initially treated with surgery had bone union without other treatment. Two out of 14 cases required a second surgery with a plate and bone graft to achieve bone union.

Conclusion

Symptoms from displaced midshaft clavicular fracture non-unions are due to related pain and dysfunctional deficits that result from displacement and shortening. According to our study, patients with a displaced midshaft clavicle fracture non-union who needed surgery achieved similar functional results as compared to patients treated in an acute setting for a displaced midshaft clavicle fracture. The median time to discharge and return to work was more than doubled in the non-union surgery group.

## Introduction

Displacement is probably the main risk factor for middle-third clavicle fractures to develop a non-union, which has created a trend in the last five years towards their surgical treatment [1].

This trend results from the positive results in the two main objectives of this treatment. One, reducing the risk of the non-union of displaced midshaft clavicle fractures, where an odds ratio (OR) of 0.22 was reported for surgical treatment in systematic reviews or an overall 1.4% rate of non-union as compared with 14.5% for non-surgical treatment [2-3]. Also, the Constant score was demonstrated to be higher after one year of surgically treated displaced midshaft fractures [4-5]. This was validated in a study on patients with workers' compensation, who had type 2b of Robinson’s classification, in which surgical treatment with plate and screws had a higher Constant score after a year, an earlier return to work, and a lower rate of non-union (as compared to those with non-surgical treatment) [6-7].

Even with these results, we cannot recommend surgical treatment of displaced middle-third clavicle fractures for all patients. The number needed to treat (NNT) to prevent one non-union has been reported from 4.8 to as high as 10.3 and 18.5 for surgical treatment [2,8-9]. Among the arguments against surgery is an elevated rate of reoperation, which is largely associated with symptoms related to plate use [10]. Other reported complications are infections of the surgical wound as well as transient neurological symptoms [11].

Questions about the cost-effectiveness of surgical treatment have been added to the discussion, which addresses the advantages and disadvantages of surgical and non-surgical treatment [12-13]. When patients are provided with the proper information, many are concerned if after non-surgical treatment, they develop a symptomatic non-union that still requires surgery, the final results will be as good as if we opted for surgical treatment from the beginning. There is a lack of information about the functional results and complications in midshaft clavicle fractures non-unions. In a recent study, 60% of patients that developed a non-union required a secondary operation and the total reported complications of surgical treatment in this group has been higher than in acute fractures [8,14].

Non-union surgical treatment results are important information to counsel patients with a displaced middle-third clavicle fracture. This study’s main objective was to compare the functional outcomes of patients with acute surgical treatment of a displaced middle-third clavicle fracture vs. patients who were surgically treated for a symptomatic non-union of a displaced middle-third clavicle fracture. Secondary objectives were to evaluate the total time out of work with each treatment and the union rate of both groups.

Our working hypothesis was that there is no difference in functional outcomes between the surgical treatment of an acute displaced middle-third clavicle fracture and the non-union surgical treatment of a displaced middle-third clavicle fracture. However, acute surgical treatment tends to lead to a patient’s earlier return to work.

## Materials and methods

**Figure 1:**
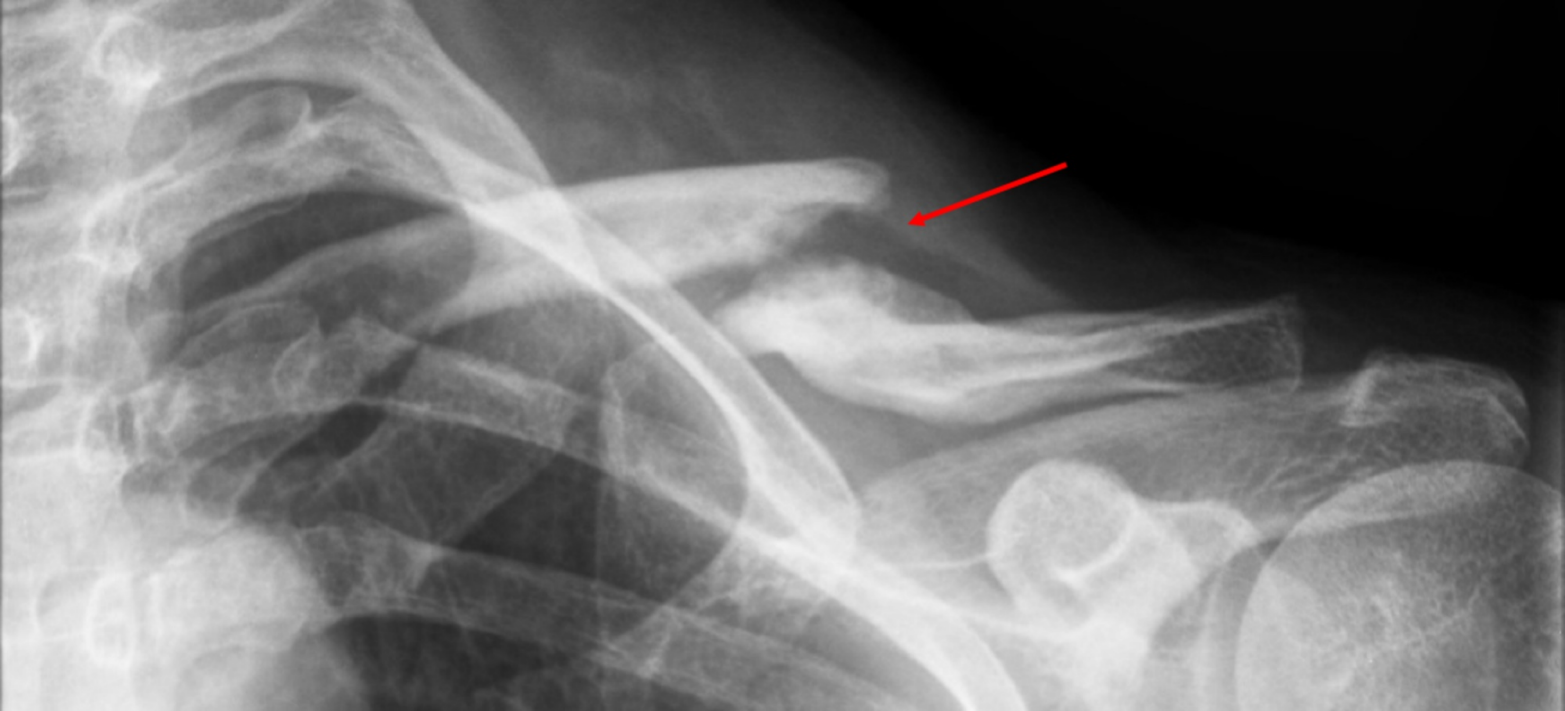
X-Ray example of a midshaft clavicle fracture non-union Red arrow: non-union

This was a case-controlled study; the cases that were considered were of consecutive patients with a displaced midshaft clavicle fracture (Robinson 2b), initially treated non-surgically but that developed a symptomatic non-union and required surgical treatment between 2012 and 2014 (Figure 1). Initial treatment considered three weeks of shoulder immobilization and physiotherapy. Symptomatic non-union treatment was considered in patients with at least four months of follow-up, as well as without X-ray evidence of bone union and persistent pain symptoms. The control group was a prospective cohort of consecutive patients with a displaced midshaft clavicle fracture, who had surgical treatment in an acute setting (<3 weeks).

The cases did not receive previous treatment for non-union issues until surgery. Both groups were surgically treated with open surgery and osteosynthesis (OTS), which included plates and screws. An iliac crest bone graft was used in the non-union group.

The inclusion criteria for both groups were:

1. Displaced midshaft clavicle fractures (Robinson 2b)

2. Surgical treatment, including open treatment and OTS

3. A CT scan during follow-up for bone union evaluation

4. Functional evaluation at least after 12 months of follow-up

The exclusion criteria were:

1. Medial or lateral clavicle fractures

2. Associated vascular or neurologic injury

3. Open fractures

4. Associated ipsilateral fractures

The variables measured included: (1) Time to discharge for return to work, measured in months; (2) Bone union confirmed by CT scan; (3) Functional results measured with the Constant score [15]. A Kaplan-Meier analysis (Log-Rank test) was done to measure the time to discharge and the return to work. A median difference test for independent variables was also done, which considered a significant difference to be p-value ≤ 0.05. For all tests, STATA v 11.2 software was used (StataCorp LP, TX, USA).

## Results

A total of 17 consecutive patients went to surgical treatment for a symptomatic non-union of a displaced middle-third clavicle fracture from years 2012 to 2014. Of them, 14 patients completed at least one-year follow-up, had a CT scan during follow-up, and went through a functional evaluation. The median time to surgery since the initial trauma of the non-union group was 5.2 months (4-15). The control group was a consecutive cohort of 18 patients, surgically treated for a displaced midshaft clavicle fracture between 2010 and 2012. Both groups included only patients with labor accidents treated in our hospital, who were under the working accidents compensation national law.

Our cases had a median follow-up of 77 months (range: 13 to 91). All those in the control group had a 12-month follow-up evaluation. The description of both groups can be observed in Table 1.

**Table 1:** Cases and control group comparison

	Non-union surgery group (Cases)	Acute surgery group (Control)	P (test)
Female	7	2	
Men	7	16	0.02 (Fisher)
Age	44.92 (±12.91)	36 (±12.91)	0.20 (Wilcoxon)
Fracture type 2B1	9	8	
Fracture type 2B2	5	10	0.23 (Fisher)

**Figure 2:**
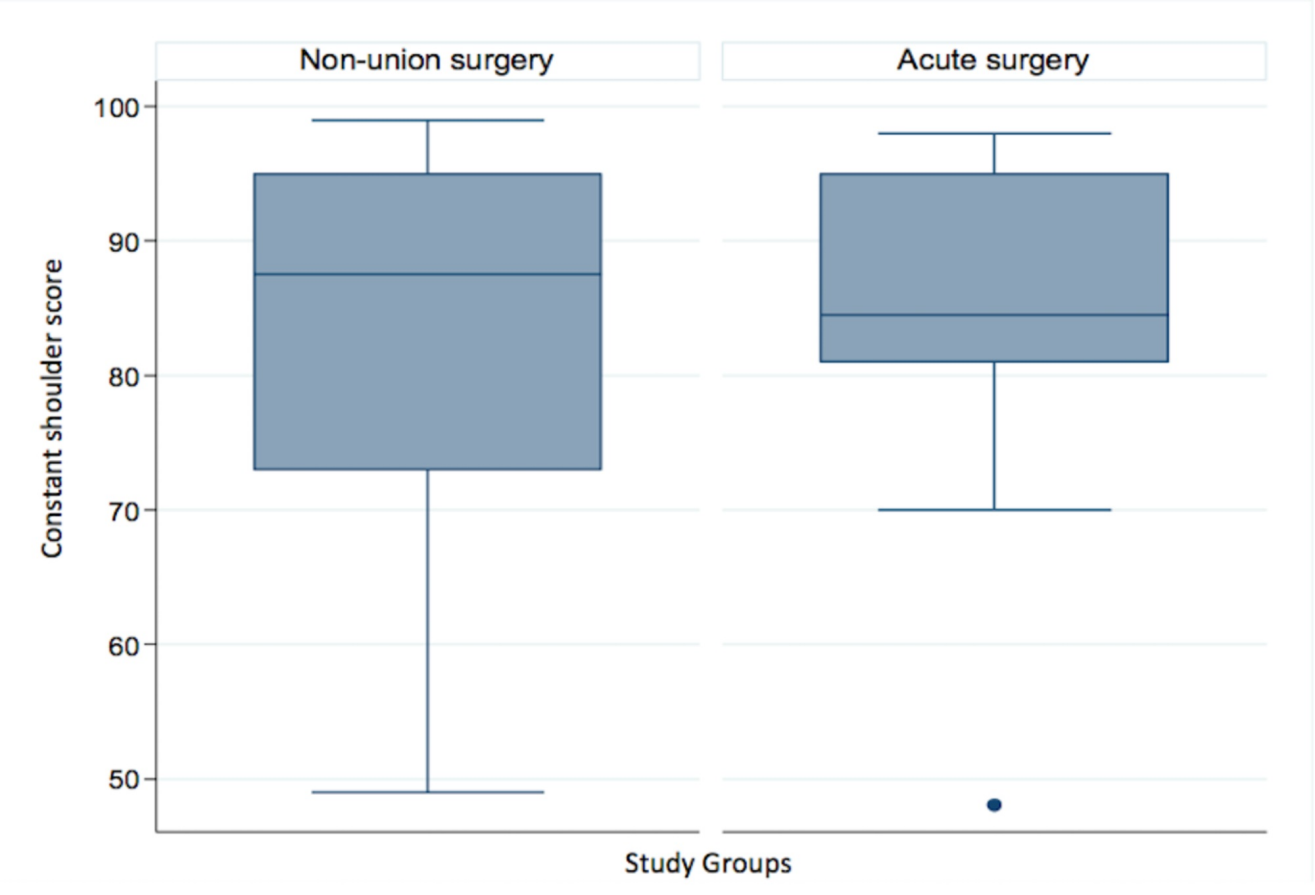
Constant score comparison between both study groups

The median Constant score at final follow-up for surgically treated non-unions was 87.5 and for the control group, it was 84.5 (p >0.05). A detailed Constant score distribution and comparison between groups can be observed in Figure 2.

**Figure 3:**
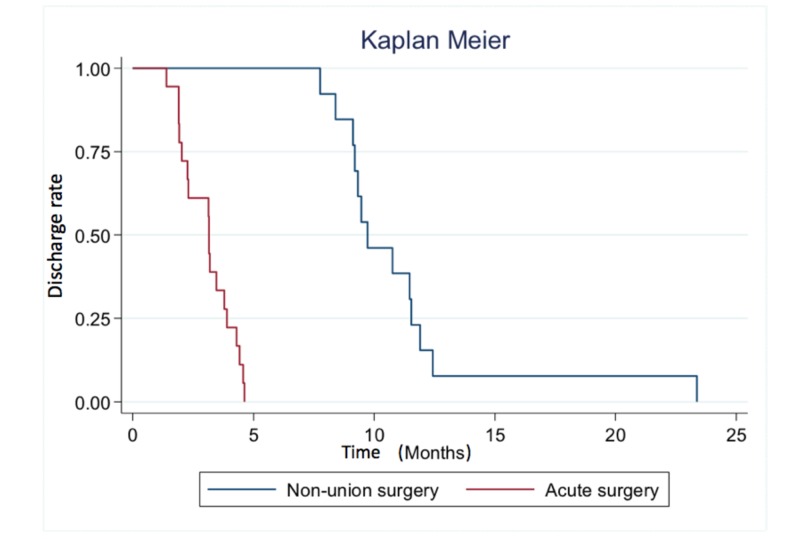
Median time to discharge to work

The median time to complete return to work since the injury date was 3.2 months in the control group and 9.7 months in the case group (p = 0.001), all being discharged from seven to 12 months after the fracture date, as seen in Figure 3. The median time to complete return to work since surgery in the non-union group was 4.9 months (2.4 - 7.6).

**Figure 4:**
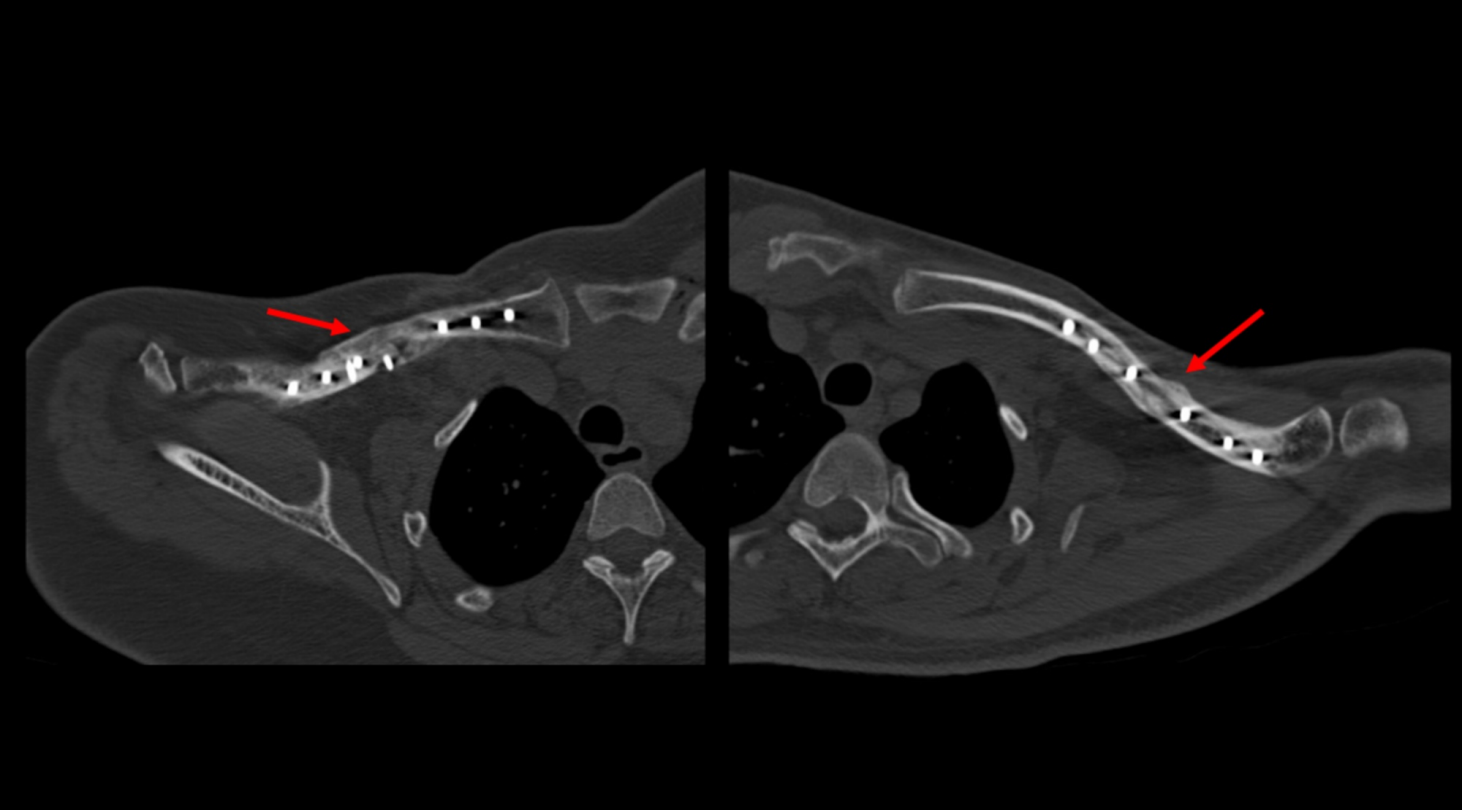
CT scans and bone union achieved after surgery CT: computed tomography Red arrow: signs of bone union

One hundred percent of those patients who were initially treated with surgery had bone union on CT scan without other treatment. However, two out of 14 cases required a second surgery with a plate and bone graft to achieve bone union. Both achieved bone union during follow-up after a second surgery. A CT scan of bone unions can be seen in Figure 4.

One patient in the acute surgical treatment group required a second surgery to remove a symptomatic plate. Both groups did not develop complications as superficial or deep infections, wound dehiscence, neurological deficits, or vascular injuries.

## Discussion

Mckee et al. reported, in a group of 30 patients with a displaced midshaft clavicle fracture with nonoperative treatment, less strength (67% vs. 82% of the contralateral shoulder) and an average Constant score of 71 with a Disabilities of the Arm, Shoulder, and Hand (DASH) score of 24.6, showing a functional deficit in these patients [16].

Symptoms from a displaced non-union midshaft clavicular fracture are due mainly to related pain from non-union problems but also from dysfunctional deficits that may result from displacement and shortening. It has been previously described that the functional impairment of these last few factors may be corrected with a later surgery [17].

In our results, Constant scores were similar at the end of the patients’ follow-up between those treated with surgery in the acute setting and those operated on later with a non-union (84.5 vs. 87.5, p = >0.05). However, surgical treatment in the non-union group showed a Constant score with wide dispersion. This prompted us to think that positive results might be less predictable than displaced midshaft clavicular fractures with surgical treatment in the acute setting.

These results differed from those reported by Potter et al. [18], with a statistically significant difference in the Constant score in favor of acute surgery (95 vs. 89 p = 0.02). Conversely, the median difference of 6 points may be clinically irrelevant [19-21]. They did not find any difference in the DASH score, patient satisfaction, or absolute strength. However, the non-union surgical treatment group had lower resistance strength at the end of the follow-up.

In a recent study by McKnight et al,, 209 patients undergoing surgical treatment for non-union had a higher rate of total complications as compared with the acute fracture group (5.26% vs. 2.28%; p = .034), a more than two-fold increased risk of any postsurgical complication (OR, 2.29 (95% CI, 1.05-5.00); p = .037) and >three-fold increased risk of a wound complication (OR, 3.22 (95% CI, 1.02-10.20); p = .046) compared with acute fractures [14].

There was a significant difference in the median time to discharge to return to work between our study group (9.7 months) and the control group (3.2 months). This may be of interest in a workers’ compensation population under labor insurance coverage and should be included in any cost-effective analysis. All patients with acute surgery were discharged for return to work before five months; however, our non-union surgery group required at least seven months from the initial trauma. A similar trend was observed in a group of 11 patients who went through a non-union surgery, where it took them more time to return to functional activities than patients surgically treated in the acute setting, but a similar single assessment numerical evaluation (SANE) score between both groups was finally obtained at the one-year follow-up [22].

We achieved bone union in all patients with acute surgery. Two patients in the study group had a non-union and plate loosening during follow-up. They both required another surgery with a plate and bone autograft, and they finally achieved bone union after one year of follow-up.

In this case-controlled study, the non-union surgical group’s functional results were like previously published results in an acute surgery setting, even though our study included patients with labor compensation [3-5,23].

This study is limited by the retrospective analysis of the non-union surgical group. Also, the small numbers of both groups and a 1:1 matching process limited the statistical analysis. Both groups did not match on gender and were not matched in other factors that may contribute to results as smoking status, although these factors have only been identified as risk factors for non-union in displaced middle-third clavicle fractures treated conservatively. The strength is the median follow-up of the non-union surgically treated group, being the longest one reported with functional results in the literature.

More prognostic studies about clavicle fracture non-union surgery are needed to advise patients whether surgical treatment is recommended for their fracture in the acute setting and what to expect if they need surgery for a non-union. Ideally, they should be high-quality prospective cohort studies, with patients enrolled at the same time point in the disease.

## Conclusions

As actual evidence does not support surgical treatment for all displaced midshaft clavicle fractures, we should know the functional results expected for those patients that developed a symptomatic non-union and needed surgical treatment. According to our study, patients with a displaced midshaft clavicle non-union fracture who needed surgery achieved similar functional results as compared to patients treated in an acute setting for a displaced midshaft clavicle fracture. Despite these results, the median time to discharge and return to work were more than doubled in the non-union surgery group.
